# Low Levels of Physical Activity and Sedentary Behavior in Adults with Intellectual Disabilities

**DOI:** 10.3390/ijerph14121503

**Published:** 2017-12-04

**Authors:** Kelly Hsieh, Thessa I. M. Hilgenkamp, Sumithra Murthy, Tamar Heller, James H. Rimmer

**Affiliations:** 1 Department of Disability and Human Development, University of Illinois at Chicago, Chicago, IL 60609, USA; smurthy@uic.edu (S.M.); theller@uic.edu (T.H.); 2 Department of Kinesiology and Nutrition, University of Illinois at Chicago, Chicago, IL 60612, USA; thessa@uic.edu; 3 School of Health Professions, University of Alabama at Birmingham, SHPB 331, Birmingham, AL 35294, USA; jrimmer@uab.edu

**Keywords:** intellectual disability, low levels of physical activity, sedentary behavior, TV watching

## Abstract

Adults with intellectual disabilities (ID) are more likely to lead sedentary lifestyles and have low levels of physical activity (LLPA). The present study investigated the prevalence of reported LLPA and time spent watching TV in adults with ID and identified the associated factors for these behaviors. The proxy informants of 1618 adults with ID completed the surveys regarding their health behaviors. Multiple logistic regressions were employed for LLPA and multiple linear regressions for time spent watching TV. About 60% of adults with ID had LLPA and average time spent watching TV was 3.4 h a day. Some characteristics and health and function variables were identified as associated factors. While engaging in community activities and involvement in Special Olympics were inversely associated with LLPA, they were not associated with time spent watching TV. Attending day/educational programs or being employed were associated with spending less time watching TV. Findings highlight differential factors associated with LLPA versus TV-watching behavior in adults with ID. Hence, a key strategy aimed at increasing physical activity includes promoting participation in social and community activities, while targeted activities for reducing sedentary behavior might focus on providing day programs or employment opportunities for adults with ID.

## 1. Introduction

Low levels of physical activity (LLPA) have long been recognized as a major risk factor for the development of metabolic syndrome, chronic health conditions, and obesity-related comorbidities in the general population [1,2]. Physical inactivity is also identified as the fourth leading risk factor for mortality, with an estimated three million deaths annually across the world [3]. More recently, sedentary behavior has also been shown to be a risk factor for type 2 diabetes, cardiovascular disease, and mortality, which may be independent of the amount of physical activity the individual performs per se [4,5,6,7]. Sedentary behavior is referred to as any waking behavior characterized by a low energy expenditure ≤1.5 metabolic equivalent units (METs) while in a sitting, reclining or lying posture (e.g., use of electronic devices—television, computer, tablet, phone, or sitting in a car, bus, or train) [8]. Such behavior has its own detrimental health effects on metabolic risk factors such as high blood glucose and elevated triglyceride levels [9,10]. Therefore, careful monitoring of physical activity and sedentary behavior are both critical for understanding risk factors associated with all-cause mortality and morbidity.

Reducing sedentary behavior may require different strategies and interventions compared to increasing physical activity. Sedentary behavior is presumably more ubiquitous, habitual, and socially-reinforced in nature and involves different motivational processes than the decision to engage in planned and effortful physical activity [11]. There is consensus that it is important to investigate determinants for each of these behaviors separately [12].

Previous studies [13,14,15,16] report that adults with intellectual disabilities (ID) have high rates (58–89%) of not meeting physical activity (PA) recommendations which stipulate that adults with disabilities, who are able to, should perform 150 min of moderate-intensity physical activity per week or 75 min a week of vigorous-intensity physical activity, or an equivalent combination of moderate and vigorous-intensity aerobic activity [17]. Insufficient physical activity has been an established health concern in this population for over a decade [14,18,19]. A number of studies have examined the determinants of not meeting PA recommendations in this population. The identified associated factors for not meeting PA guidelines have included personal (e.g., severity of ID, age, sex, obesity, living in care, mobility limitation, health problems), and social-environmental factors (e.g., community participation, social support residential settings, access to facilities, neighborhood safety) [15,20,21,22]. In addition, identified predictors for low levels of physical activity in the general population include older age, having immobility, epilepsy, fecal incontinence, lack of day opportunities, and living in congregated settings [23]. Hence, these factors may need to be taken into account while investigating associated factors for low levels of physical activity in adults with ID. 

Sedentary time (the amount of time spent in any sedentary behaviors) has only recently received attention in research focused on individuals with ID. It has been used as a covariate to explain intervention and health outcomes [24,25,26], as well as a secondary outcome measure in physical activity studies involving adults with ID [27,28,29]. With the exception of one study that examined sedentary behavior as a primary outcome in adolescents and young adults with Down syndrome [30], little research exists on what factors are associated with sedentary time in adults with ID. Because of the use of varied outcome measures (e.g., different questionnaires, pedometers, accelerometers, and cut-off values) and small sample sizes in most of these studies, there is a lack of clarity on the prevalence of sedentary behavior in a large cohort of individuals with ID. 

A systematic review on sedentary behavior in older adults in the general population showed that sedentary time increased with age, although it decreased with retirement. Health and well-being were related to less sedentary time, while obesity was related to more sedentary time [31]. A population-based study noted that people who reported three hours or more of television viewing a day had a two-fold higher risk of mortality than those reporting less than one hour a day after adjusting for individual characteristics and health risk behaviors [32]. Thus, amount of time spent watching TV might be an important index for examining sedentary behavior in adults with ID. 

Social-environmental factors that relate to sedentary behavior have received little attention. Possible determinants may be mode of transport, type of housing, cultural opportunities, neighborhood safety, and availability of places to rest [31]. A more recent study found that a sense of community belonging was associated with less sedentary behavior [33]. The need for more research on determinants of sedentary behavior is identified in the most current reviews and knowledge updates [12,31,34,35]. While information from the general population might be useful to inform research on determinants of sedentary behavior in individuals with ID, personal and contextual factors need to be identified for this population as they may be very different from the general population. To date, the available information on determinants of sedentary behavior in individuals with ID is scarce. One study involving 96 adults with Down syndrome found a relationship between being of older age and female, and increased sitting time [30]. These correlations need to be confirmed in other samples of people with ID and the list of associated factors needs to be expanded with other potential correlates.

To shed light on the association between LLPA and sedentary behavior and their associated risk factors in adults with ID, the present study examined the: (1) prevalence of LLPA and average time spent in sedentary behavior in adults with ID; (2) association between the time spent in sedentary behavior (TV watching) and LLPA; and (3) associated factors (demographic, healthand function, as well as social-environmental factors) for LLPA and sedentary behavior (time spent watching TV).

## 2. Materials and Methods

### 2.1. Participants

Participants in the present study were the baseline cohort of a 9-year ongoing longitudinal study on health risk factors in U.S. adults with ID (Longitudinal Health and Intellectual and Developmental Disability Study) [36]. Most of the study participants were receiving social or clinical services from community agencies (e.g., residential, daytime services, or case management, managed-care organizations) that support individuals with intellectual and developmental disabilities across the 50 states. A total 1618 participants were in the baseline group. Intellectual disability-related conditions and level of ID were recorded and are presented in the demographic table in the results section.

### 2.2. Procedure

We recruited family members or primary caregivers of adults with ID as informants by inviting them at Special Olympics events, posting recruitment information in various avenues (e.g., Facebook, newsletter advisements, and recruiting materials distributed at conferences), collaborating with managed-care organizations, and working with agency staff from various community service agencies. Baseline data were collected from informants between 2010 and February 2011. A mixed-method (mail and online surveys) data collection procedure was used; hence informants could complete a paper or online survey based on their preference. The University institutional review board approved the present study involving human subjects. The survey along with a cover letter and a subject information letter that served as informed consent for the study was sent to the informants. A total of 2841 surveys (2182 paper and 659 online) were distributed, and 1618 surveys (1187 paper and 431 online) were completed and returned, with an overall response rate of 57%. The response rates for the paper survey and online survey were 65% and 54%, respectively. Almost half the surveys were completed by parents (48.3%), 22.1% by health care providers, 14% by residential or day program staff, 12.7% by relatives other than parents or non-related live-in caregivers, and 2.9% by others or the adults with ID themselves.

The test–retest reliability of survey questions ranged from acceptable to very high. For the categorical questions, the test–retest reliability (k-statistic) ranged from 0.68 to 0.95, and for the interval questions, the test–retest reliability intraclass correlation coefficient) ranged from 0.75 to 0.94. A detailed description of the survey development was described in a previous paper [36].

### 2.3. Measures

The outcome measures in the present study were LLPA and time spent watching TV. The independent variables included three factors: (1) demographic; (2) health and function; and (3) social-environmental factors.

Low levels of physical activity (LLPA). Participants who were reported that they never/rarely participated in any type of PA were classified as participating in no PA. Participants who were reported that they participated in moderate or vigorous physical activities once or twice a week were classified as participating in little PA. We defined low levels of PA as participating in little or no PA. Informants were asked two separate questions: “How many days a week does he/she do moderate physical activities for at least 30 min on average?”, and “How many days a week does he/she do vigorous activities for at least 20 min?”. The responses included “never/rarely”, “1 or 2 times a week”, “3 times a week”, and “4 or more times a week”.

Time spent watching TV. Sedentary behavior was assessed by number of hours spent on TV watching on an average day. Ten responses ranged from 0 through 9 or more hours.

Demographic factors. Demographic information included age, sex, race, level of ID (borderline, mild or moderate, and severe or profound), and intellectual disability-related conditions (Down syndrome, cerebral palsy, autism spectrum disorder, ID only, and other conditions).

Health and function factors. Health and function were described by 10 different aspects. The first was informant-rated health status. Informant-rated health status was collected using a 5-point Likert scale ranging from 1 “poor” to 5 “excellent”. The second aspect was with respect to the days with activity limitation. The number of days that usual activity was limited due to poor physical or mental health during the past 30 days was calculated. The third aspect was the number of chronic health conditions. Informants were asked to check whether the person with ID had a diagnosis of 36 health conditions (27 physical health and 9 mental health conditions). The fourth aspect was obesity. Informant-reported body weight and height were used to calculate body mass index (BMI) by using the formula (weight in pounds/(height in inches)^2^) × 703. Based on the Centers for Disease Control and Prevention criteria, obesity was defined as BMI ≥ 30.0 kg/m^2^. The fifth aspect was depression. Whether the person with ID had a diagnosis of depression was coded as (1) Yes or (0) No. The sixth aspect was use of psychotropic medications. Using medications for anxiety, bipolar disorder, obsessive-compulsive disorder, or schizophrenia was coded as using psychotropic medications (1) Yes or (0) No. The seventh aspect was related to epilepsy/seizure disorders. Whether the person with ID had a diagnosis of epilepsy/seizure disorder was coded as (1) Yes or (0) No. The eighth aspect was urinary incontinence. Whether the person with ID had a diagnosis of urinary incontinence was coded as (1) Yes or (0) No. Aspect nine was related to falls. Informants were asked, “How many falls has the person with ID experienced in the past 12 months?”. A fall was defined as “a sudden unintentional change in position causing an individual to land at a lower level, on an object, the floor, or the ground, other than as a consequence of a sudden onset of paralysis, epileptic seizure, or overwhelming external force” [37]. Participants who experienced one or more falls in the past 12 months were grouped as having falls. The final aspect was mobility limitation. Use of any walking aids (i.e., a cane, crutches, a walker, or a wheelchair) was coded as having a mobility limitation (1) Yes or (0) No.

Social-environmental factors. Social-environmental factors included day/educational programs or employment participation status, residential type, residential location, social participation, and Special Olympics involvement. Day/educational program or employment participation status was defined as part-time or full-time participation in any day or educational program or employment Residential type was divided into three categories: own homes, family homes, and foster/group homes. Residential location was classified into rural and urban based on the participant’s zip code using Census 2000 definitions. The file for urban/rural classification was downloaded for Zip Code Tabulation Areas (ZCTAs). The file contains population numbers for three types of areas: urbanized area, urban cluster, and rural. The data were collapsed into one dichotomous variable. Zip codes with populations in the urbanized area or urban cluster were classified as urban and the remaining sample as rural. Participant zip codes were matched to the ZCTAs so that they contained the urban/rural classification. Social participation assessed the engagement in four types of social activities (talking to family members or friends on the phone, visits with family members, visits with friends, and going to movies, sports events, and clubs) in the last month. All items were scored on a 4-point Likert scale, ranging from 1 “not at all” to 4 “2 or more times a week”. The total scale score ranged from 4 through to 16. Special Olympics involvement was assessed by the number of Special Olympics events (ranged from 0 “no event” to 4 “4 or more events”) that participants with ID participated in during the past 12 months. 

### 2.4. Data Analysis

Descriptive statistics were used to examine the prevalence of LLPA and time spent watching TV. Chi-square tests were used to examine the group differences in the proportions of categorical variables. Independent sample *t*-tests and analysis of variance (ANOVA) tests were used to examine group mean differences in continuous variables. Post hoc comparisons using the Tukey Honestly Significantly Difference (HSD) test were employed when significant ANOVA tests were presented. A significance level at a *p* value of 0.05 was used for these tests. We employed the purposeful selection process [38]. The process included a series of univariate binary logistic regressions for the dichotomous dependent variable (LLPA) and a series of simple linear regressions for the continuous dependent variable (time spent watching TV) to identify potential risk factors. The independent variables including participant demographic factors (age, sex, level of ID, ID-related conditions); health and function factors (informant-rated health status, days of activity limitation, number of chronic conditions, obesity, depression, use of psychotropic medication, epilepsy/seizure, urinary incontinence, falls in the past 12 months, and mobility limitation); and social-environmental factors (day/educational program or employment participation status, residential type, residential location, social participation, and Special Olympics involvement) were entered separately into a univariate regression model for each outcome measure. Using a traditional *p* value of 0.05 may fail to identify variables of known importance. Therefore, to identify potential covariates and confounders, any independent variables in a univariate regression model with a cut-off point of *p* < 0.20 [39,40] on the Wald test were included in the multiple regression models. 

As a result, all independent variables met the cut-off criteria in the univariate analysis and were included in the final multiple logistic regression models for LLPA. Five variables (number of chronic conditions, depression, falls, residential location, social participation, and Special Olympics involvement) were excluded in the final multiple regression models for time spent watching TV. Although age did not meet the inclusion criteria (*p* < 0.020) in the univariate analysis for time spent watching TV, it is included based on the literature review. Level of ID was included in Model I and was excluded in Model II multiple regressions due to unknown and missing data regarding level of ID (*n* = 437). The multiple logistic and linear regression models were conducted using the block entry method to examine associated factors for the outcome variables (LLPA and time spent watching TV). We assessed collinearity using car (Comparison to Applied Regression) package 2.1-5, vif function in R 3.4.1 (R Foundation for Statistical Computing, Vienna, Australia) [41,42] to calculate the generalized variance inflation factor (GVIF) among independent variables in the multiple logistic models. For the final multiple linear regression models, VIFs were calculated to assess collinearity. All the data analyses were conducted using IBM SPSS Statistics for Windows, Version 24.0 (IBM Corp., Armonk, NY, USA) [43].

## 3. Results

### 3.1. Descriptive of Study Participants

The study participants (*N* = 1618) consisted of 893 (55.2%) men and 725 (44.8%) women, with a mean age of 37.67 (*SD* = 14.39) years, ranging from 18 to 86 years. Most of the participants were White (89.2%) followed by Black (6.2%), American Indian/Alaskan Native (1.6%), Hispanic (1.5%) and Asian/Pacific Islander (1.5%). More than half of the participants had mild or moderate ID (52.4%); 12.4% had borderline ID; and 8.2% had severe or profound ID. Twenty-seven percent of participants (*n* = 437) were reported to have a missing or an unknown level of ID. One quarter of participants (25%) had Down syndrome, 12.2% had autism, and 12.7% had cerebral palsy. More than half of the participants (56.4%) lived with family, whereas 29.4% lived on their own and 14.2% lived in a foster or group home. A majority of the participants lived in urban areas (85.5%). Approximately 87% of the participants participated in a day/educational program or were employed. More than half of the participants (53.7%) were reported as having very good or excellent health. Over one-third of the participants were obese; 15.4% had depression; 21.3% used psychotropic medication; 19% had seizure disorder; and 15% had mobility limitation (see Table 1).

### 3.2. Prevalence of Low Levels of Physical Activity

Almost 60% of the participants had LLPA. Significantly more adults with ID aged 60 and over had LLPA (80.1%) as compared to those aged 18–39 years old (53.5%) and 40–59 years old (66.0%), *X*^2^ (2, *N* = 1618) = 48.28, *p* < 0.001. Significantly more females (65.2%) had LLPA as compared to males (55.5%), *X*^2^ (1, *N* = 1618) = 15.53, *p* < 0.001. Significantly more adults with severe or profound ID had LLPA (74.2%) as compared to those with borderline (54.2%) and mild or moderate ID (57.5%) *X*^2^ (2, *N* = 1181) = 15.28, *p* < 0.001. The prevalence of LLPA was significantly higher in adults with ID and cerebral palsy (77.2%) compared to those with Down syndrome (56.5%), autism spectrum disorder (51.4%), and ID only or other diagnoses (58.7%), *X*^2^ (3, *N* = 1487) = 31.33, *p* < 0.001. Adults with ID who lived in a group or foster home (71.4%) had significantly more LLPA than those who lived with family (55.8%) and those who lived independently (62.4%), *X*^2^ (2, *N* = 1547) = 19.21, *p* < 0.001. 

### 3.3. Time Spent Watching TV

Overall, adults with ID aged 18 and older spent more than three hours a day (*M* = 3.42, *SD* = 2.13) watching TV. Over three-fifths of the participants (61.5%) spent three or more hours watching TV on an average day. About 40% of participants spent 4 h or more watching TV. Men with ID spent more time a day watching TV (*M* = 3.55, *SD* = 2.17) than women with ID (*M* = 3.26, *SD* = 2.04), *t*(1563) = −2.64, *p* = 0.004. No significant differences in time spent watching TV among the three age groups and ID related conditions were noted. There was a statistically significant difference in time spent watching TV between levels of ID as determined by one-way ANOVA, *F*(2, 1154) = 7.08, *p* = 0.001. Post hoc comparisons using the Tukey HSD test indicated that the time spent watching TV was statistically higher in the group with borderline ID (*M* = 3.77, *SD* = 2.15) than in the group with mild or moderate ID (*M* = 3.37, *SD* = 2.00) and the group with severe or profound ID (*M* = 2.89, *SD* = 2.20). The participants with mild and moderate ID also spent significantly more time watching TV than those participants with severe or profound ID. There was also a statistically significant difference in time spent watching TV among residential types as determined by a one-way ANOVA, *F*(2, 1498) = 10.49, *p* = 0.00. The Tukey HSD test indicated that the time spent watching TV was statistically higher for those living in their own homes (*M* = 3.45, *SD* = 2.25) or family homes (*M* = 3.58, *SD* = 2.11) than those living in foster/group homes (*M* = 2.84, *SD* = 1.94). There was no difference in time spent watching TV between own homes and family homes or between urban and rural locations. 

### 3.4. Association between Low Levels of Physical Activity and Sedentary Behavior

Adults with ID who were in the LLPA group spent more time watching TV (*M* = 3.57, *SD* = 2.22) than those who were in the non-LLPA group (*M* = 3.21, *SD* = 1.95), *t*(1559) = −3.33, *p* = 0.001. Male participants who were in the LLPA group spent significantly more time watching TV (*M* = 3.76, *SD* = 2.25) than those who were in the non-LLPA group (*M* = 3.29, *SD* = 2.04), *t*(865) = −3.21, *p* = 0.001. For female participants, even though those who were in the LLPA group spent more time watching TV (*M* = 3.37, *SD* = 2.17) than those who were in the non-LLPA group (*M* = 3.08, *SD* = 1.80), the differences were not significant. The Pearson correlation for LLPA and time spent watching TV was significant, (*r* = 0.08, *p* = 0.001).

### 3.5. Results of Univariate Regressions: Low Levels of Physical Activity and Time Spent Watching TV

Table 2 presents a summary of univariate regressions based on a series of univariate logistic regressions and linear regressions. Significant variables (*p* < 0.02) in the univariate logistic regressions for LLPA included age 40–59 years (odds ratio (*OR*) = 1.69, 95% confidence interval (*CI*) = 1.35–2.11) and 60 years and above (*OR* = 3.53, 95% *CI* = 2.29–5.45), being male (*OR* = 0.67, 95% *CI* = 0.55–0.82), having Down syndrome (*OR* = 2.64, 95% *CI* = 1.77–3.93), or severe or profound ID (*OR* = 2.43, 95% *CI* = 1.51–3.93), informant-rated health status (*OR* = 0.61, 95% *CI* = 0.54–0.69), days of activity limitation (*OR* = 1.05, 95% *CI* = 1.03–1.08), number of chronic conditions (*OR* = 1.08, 95% *CI* = 1.04–1.12), obesity (*OR* = 1.42, 95% *CI* = 1.15–1.76), depression (*OR* = 1.38, 95% *CI* = 1.04–1.83), epilepsy/seizure disorder (*OR* = 1.51, 95% *CI* = 1.16–1.97), urinary incontinence (*OR* = 2.50, 95% *CI* = 1.73–3.62), falls (*OR* = 1.61, 95% *CI* = 1.26–2.06), mobility limitation (*OR* = 4.66, 95% *CI* = 3.22–6.75), not participating in day/educational programs or employment (*OR* = 1.27, 95% *CI* = 0.94–1.74), living in family homes (*OR* = 0.76, 95% *CI* = 0.60–0.96), living in foster or group homes (*OR* = 1.51, 95% *CI* = 1.06–2.14), social participation (*OR* = 0.86, 95% *CI* = 0.83–0.89), and Special Olympics involvement (*OR* = 0.80, 95% *CI* = 0.76–0.85).

Significant variables associated with time spent watching TV in the univariate linear regressions included demographic factors: male sex (unstandardized beta coefficient (*B*) = 0.28, 95% *CI* = 0.07–0.50), ID related conditions (cerebral palsy, *B* = −0.21, 95% *CI* = −0.55–0.13; autism spectrum disorder, *B* = −0.29, 95% *CI* = −0.63–0.06), level of ID (mild or moderate, *B* = −0.40, 95% *CI* = −0.72–−0.09; severe or profound, *B* = −0.88, 95% *CI* = −1.34–−0.41); health and function factors: informant-rated health status (*B* = −0.02, 95% *CI* = −0.04–0.00), days of activity limitation (*B* = 0.02, 95% *CI* = −0.01–0.04), obesity (*B* = 0.52, 95% *CI* = 0.30–0.74), use of psychotropic medications (*B* = −0.44, 95% *CI* = −0.70–−0.19), epilepsy/seizure disorder (*B* = −0.19, 95% *CI* = −0.46–0.08), urinary incontinence (*B* = −0.63, 95% *CI* = −0.96–−0.29), mobility limitation (*B* = −0.22, 95% *CI* = −0.52–0.08); and social-environmental factors: not participating in day/educational program or employment (*B* = 0.99, 95% *CI* = 0.67–1.30), and residential type (foster/group home, *B* = −0.61, 95% *CI* = −0.96–−0.27).

### 3.6. Low Levels of Physical Activity and Associated Factors

Table 3 presents the adjusted odds ratios (AOR) from the results of the multiple logistic regressions for LLPA with level of ID included in Model I (*N* = 926) and excluded in Model II (*N* = 1228). In Model I where the variable of level of ID was included, male sex (*AOR* = 0.70, 95% *CI* = 0.52–0.95), cerebral palsy (*AOR* = 1.93, 95% *CI* = 1.09–3.44), informant-rated health status (*AOR* = 0.72, 95% *CI* = 0.59–0.89), obesity (*AOR* = 1.60, 95% *CI* = 1.17–2.19), depression (*AOR* = 1.67, 95% *CI* = 1.02–2.71), mobility limitation (*AOR* = 2.34, 95% *CI* = 1.23–4.47), not participating in day/educational program or employment (*AOR* = 0.60, 95% *CI* = 0.36–0.98), and social participation (*AOR* = 0.86, 95% *CI* = 0.82–0.91) were significant. In Model II (*N* = 1228) where the variable of level of ID was excluded, age 60 years and above (*AOR* = 2.10, 95% *CI* = 1.12–3.92), cerebral palsy (*AOR* = 1.94, 95% *CI* = 1.21–3.13), informant-rated health status (*AOR* = 0.80, 95% *CI* = 0.68–0.95, days of activity limitation (*AOR* = 1.04, 95% *CI* = 1.01–1.08), obesity (*AOR* = 1.58, 95% *CI* = 1.21–2.06), mobility limitation (*AOR* = 2.32, 95% *CI* = 1.41–3.80), social participation (*AOR* = 0.88, 95% *CI* = 0.84–0.92), and Special Olympics involvement (*AOR* = 0.92, 95% *CI* = 0.85–0.98) were significant. The findings indicate that adults with ID who are female, have cerebral palsy, have poorer health, have obesity and mobility limitation, are in a day/educational program or employment, and engage in less community activities are more likely to have LLPA when level of the ID was included in Model I. When the level of ID was not included in Model II, adults with ID who are 60 years and older, have cerebral palsy, poorer health, more days of activity limitation, obesity and mobility limitation, and engage in less community activities and Special Olympics events are more likely to have LLPA. 

### 3.7. Time Spent Watching TV and Associated Factors

Table 4 presents the beta coefficient (*B*) from the results of the multiple linear regressions for time spent watching TV with level of ID included (*N* = 970) in Model I and not included (*N* = 1292) in Model II. The results for Model I indicated that the significant variables associated with time spent watching TV included: male sex (*B* = 0.34, 95% *CI* = 0.09–0.59), level of ID (severe or profound, *B* = −0.76, 95% *CI* = −1.26–−0.26), health and function factors: obesity (*B* = 0.48, 95% *CI* = 0.21–0.75), epilepsy/seizure disorder (*B* = −0.35, 95% *CI* = −0.68–0.01); and social-environmental factors: not participating in day/educational program or employment (*B* = 1.39, 95% *CI* = 0.98–1.79), and residential type (family home, *B* = 0.35, 95% *CI* = 0.03–0.66 and foster/group home, *B* = −0.51, 95% *CI* = −0.92–−0.09).

The results for Model II, in which the level of ID was not included, showed similar results as when level of ID was included except that use of psychotropic medications was significant, and epilepsy/seizure disorder and living in family home were not significant. The following variables were significant in Model II: male sex (*B* = 0.31, 95% *CI* = 0.08–0.53); health function factors: obesity (*B* = 0.44, 95% *CI* = 0.20–0.68), use of psychotropic medications (*B* = −0.30, 95% *CI* = −0.59–0.02); and social-environmental factors: not participating in day/educational program or employment (*B* = 1.14, 95% *CI* = 0.78–1.49), and residential type (foster/group home, *B* = −0.46, 95% *CI* = −0.83–−0.08).

## 4. Discussion

The results of this study show that about 60% of adults with ID had low levels of physical activity and about one quarter did not or rarely engage in any moderate or vigorous physical activities. Almost 62% of the participants spent over three hours a day watching TV. It is difficult to compare the prevalence of LLPA with other studies of people with ID because of the differences in outcome variables and measurement methods used. Physical activity measured with questionnaires, pedometers, or accelerometers yielded values of 63% to 94% of older adults with ID not meeting the physical activity guidelines of 150 min of moderate or 75 min of vigorous PA across the week [14,21,28,44]. Our finding was that 60% of adults with ID had LLPA, which is close to the range found in previous studies. This confirms the need for promoting higher levels of physical activity among adults with ID while decreasing sedentary behavior (i.e., TV viewing). 

Overall, the findings indicated that personal demographic and health factors and social-environmental factors were important determinants of LLPA and sedentary behaviors. We also found that specific variables were differentially associated with LLPA versus sedentary behaviors. For LLPA, when the level of ID is included in the regression model, being female, having cerebral palsy, obesity, depression, and mobility limitation were associated with LLPA, while having a better subjective health status, not participating in day/educational programs or employment and engaging more in community activities participation were inversely associated with LLPA. When the level of ID was not included in the regression model, being older, having cerebral palsy, more days of activity limitation, obesity, and mobility limitation were associated with LLPA, while having a better subjective health status, engaging in more community activities and participating in more Special Olympics events were inversely associated with LLPA. The association between participation in day/educational program or employment and participation in Special Olympics events with LLPA is not consistent in the two multivariable models. Participation in day/educational programs or employment is associated with LLPA when the level of ID was included in the multivariable model; however, it was no longer associated with LLPA when the level of ID was excluded. Therefore, further research on the relationship between participation in day program or employment and physical activity is needed. Participation in Special Olympics events was associated with not having LLPA only when level of ID was excluded in the multivariable model. The possible explanation is that participation in Special Olympics does not require participants to report level of ID. Hence, most Special Olympics participants might have missing data on level of ID and therefore, they were more likely to be excluded from Model I. Participation in Special Olympics events for adults with ID may increase the amount of physical activity. Engaging more community activities is associated with not having LLPA in both models. Hence, for adults with various levels of ID participation in more community activities may improve their physical activity. 

Regarding sedentary behavior as measured by time spent watching TV, when the level of ID was included in the regression model, increased time in watching TV was associated with being male, having obesity, not participating in a day/ educational program or not being employed, and living in a family home, while having severe/profound intellectual disability, epilepsy/seizure disorder, and living in a foster/group home were inversely associated with time spent watching TV. When the level of ID was not included in the regression model, the associated factors for increasing time in watching TV were similar, except having epilepsy/seizure disorder and living in a family home were no longer significant. However, reporting use of psychotropic medication was inversely associated with time spent watching TV. The difference in associated factors for LLPA and time spent watching TV confirmed that these were two different behaviors that required different approaches and strategies. However, those who were in the LLPA group spent more time watching TV than those who were in the non-LLPA group. Hence, increasing engagement in physical activities might also reduce sedentary behaviors. 

Our findings indicate that lower social participation and less Special Olympics involvement were associated with LLPA regardless of where adults with ID live after taking personal characteristics, health, and function factors into account. These findings parallel those of a previous study [21] reporting that individuals with ID who participate more in the community activities were more likely to be physically active. 

Increasing participation in physical activity among adults with ID requires that the activities are enjoyable and can be easily incorporated into their daily routine [45]. Evidence-based health promotion programs such as Health Matters [46,47] and the Bergstrom Study [48] have been able to increase physical activity in adults with ID. These programs need to be executed by personnel in community settings with attention to accessibility and availability features considered prior to implementation. Walking with a friend, gardening, Zumba and other forms of dance-based exercise, warm water aquatic classes, and yoga are a few examples of activities that many people who participate in them find them to be extremely enjoyable. In terms of reducing sedentary behavior, engaging in all kinds of community activities such as outings with friends and families, bowling, parties, park district activities, and Special Olympics events can also increase physical activity and reduce sedentary time. The current focus for interventions to reduce sedentary time rely mostly on interrupting periods of sitting, or replacing sedentary time with light-intensity activities [34,35]. Therefore, incorporating enjoyable physical activities even during TV commercials can break up long sitting time and may be one initial way to promote small increments in physical activity.

In comparing the determinants of sedentary behavior in the present study to those evaluated in a systematic review of older adults in the general population, some noticeable differences and similarities can be seen [31]. In this study and in other studies, men with ID appeared to spend more time watching TV than women, while in older adults these results are inconsistent [31]. This emphasizes the importance of investigating gender differences with more objective methods and in intervention studies. Type of living arrangements should also be taken into account when designing intervention programs for individuals with ID, especially for those living on their own. 

Obesity was associated with both LLPA and more time spent watching TV. In our previous study, we found over 38% of the current study participants with ID were obese, and women with ID (43%) had a higher rate of obesity than men with ID (34%) [26]. Therefore, a further investigation of the interactions among obesity, physical activity, and sedentary behavior is needed for people with ID.

This study has several strengths and limitations. Strengths include the large sample size and the wide range of subgroups (e.g., ID related conditions, level of ID, residential type), health and function, and social-environmental factors measured in the study. It allows us to examine group differences and their impact on LLPA and sedentary behavior. Another strength is the use of a multivariable approach to explore factors associated with LLPA and sedentary behavior in a more comprehensive way.

Study limitations include the use of an informant-based questionnaire to measure physical activity and sedentary time, which is subject to recall bias and socially desirable answers. Caution must be taken when interpreting these results as the measures were based on proxy reporting, which is not the ideal method to measure physical activity, and objective measurements were not used. The recruitment and consent strategy could have led to biases within this study; those at higher risk of developing chronic health conditions were more likely to complete the informant-based questionnaire. Objective measures provide a better estimate of the amount of physical activity and the time spent sitting, but subjective measures are recommended to describe the sedentary or physical activity behavior more qualitatively, including describing the type of behavior such as TV viewing. Another limitation is that we did not measure all sedentary behavior such as playing computer/video games, watching online movies/TV shows, or searching the internet. Furthermore, the sample was not representative of the U.S. population in terms of ethnicity or race, which limits the generalizability to other subgroups than white individuals with ID. Due to the recruitment strategies, the sample had some bias towards participants of Special Olympics (about 45% of the sample), which likely entails a more active portion of the population of individuals with ID. 

## 5. Conclusions

This exploratory study examined the associated factors for LLPA and sedentary time in a large cohort of adults with ID. Whereas the prevalence of LLPA was in line with previous research, sedentary behavior as measured by TV viewing time found that a large percentage of adults with ID spent three or more hours a day watching TV. Since TV viewing has been shown to be a good proxy for sedentary behavior and an independent risk factor for cardiovascular disease and early mortality, it is of concern that almost 62% of the participants were reported to be watching TV three hours a day. The associated factors we identified in this study are important to take into account in the design of intervention studies, and future research needs to confirm the relationships of these associated factors with objective measures of physical activity and sedentary behavior. The findings may also imply that promoting adults with ID in engaging in more community activities and participating in community employment may be a way to improve physical activity and reduce sedentary behavior. There is also a need to explore other sedentary behaviors in adults with ID to advance future research, interventions, policies, and practices. 

## Figures and Tables

**Table 1:** Participant demographics and characteristics (*N* = 1618). ID = intellectual disabilities; ref = a reference group.

Variables	*N*	Total Mean ± SD or *n* (%)
**Demographic Factors**		
Age (years)	1618	37.67 ± 14.39 (Range: 18–86)
18–39 (ref)		962 (59.5)
40–59		515 (31.8)
≥60		141 (8.7)
Sex	1618	
Female		725 (44.8)
Male		893 (55.2)
ID related conditions	1487	
ID only/others		745 (50.1)
Down syndrome		372 (25.0)
Cerebral palsy		189 (12.7)
Autism spectrum disorder		181 (12.2)
Level of ID	1618	
Borderline		201 (12.4)
Mild or moderate		848 (52.4)
Severe or profound		132 (8.2)
Unknown or missing		437 (27.0)
**Health and Function Factors**		
Informant-rated health status	1599	
Poor		13 (0.8)
Fair		127 (7.9)
Good		599 (37.5)
Very good		610 (38.1)
Excellent		250 (15.6)
Days of activity limitation	1575	1.84 ± 4.95 (Range: 0–30)
Number of chronic conditions	1618	3.11 ± 2.91 (Range: 0–26)
Obesity	1523	
Yes		578 (38.0)
No		945 (62.0)
Depression	1617	
Yes		249 (15.4)
No		1368 (84.6)
Use of psychotropic meds	1618	
Yes		344 (21.3)
No		1274 (78.7)
Epilepsy/seizure disorder	1617	
Yes		307 (19.0)
No		1310 (81.0)
Urinary incontinence	1617	
Yes		175 (10.8)
No		1442 (89.2)
Falls	1517	
Yes		373 (24.6)
No		1144 (75.4)
Mobility limitation	1587	
Yes		238 (15.0)
No		1349 (85.0)
**Social-Environmental Factors**		
Day/educational program or employment	1595	
Yes		1394 (87.4)
No		201 (12.6)
Residential type	1547	
Own home		455 (29.4)
Family home		872 (56.4)
Foster/group home		220 (14.2)
Residential location	1583	
Rural		230 (14.5)
Urban		1353 (85.5)
Social participation		10.43 ± 3.03 (Range: 2–16)
Special Olympics involvement		1.45 ± 1.76 (Range: 0–4)

Note. For the informant-rated health status variable, the percentages do not add to 100% because of rounding.

**Table 2:** Summary of univariate regressions for LLPA and time spent watching TV.

Variables	LLPA	Time Spent Watching TV
	*OR* (95% *CI*)	*B* (95% *CI*)
**Demographic Factors**		
Age (years)		
18–39 (ref)		
40–59	1.69 (1.35–2.11) ***	−0.13 (−0.36–0.10)
≥60	3.53 (2.29–5.45) ***	−0.02 (−0.40–0.37)
Sex (male)	0.67 (0.55–0.82) ***	0.28 (0.07–0.50) **
ID related conditions		
ID only/others (ref)		
Down syndrome	2.64 (1.77–3.93) ***	−0.01 (−0.28–0.26)
Cerebral palsy	0.82 (0.57–1.17)	−0.21 (−0.55–0.13) ^¥^
Autism spectrum disorder	1.10 (0.85–1.41)	−0.29 (−0.63–0.06) ^¥^
Level of ID		
Borderline (ref)		
Mild or moderate	1.15 (0.84–1.56)	−0.40 (−0.72–−0.09) *
Severe or profound	2.43 (1.51–3.93) ***	−0.88 (−1.34–−0.41) **
**Health and Function Factors**		
Informant-rated health status	0.61 (0.54–0.69) ***	−0.02 (−0.04–0.0) ^¥^
Days of activity limitation	1.05 (1.03–1.08) ***	0.02 (−0.01–0.04) ^¥^
Number of chronic conditions	1.08 (1.04–1.12) ***	−0.00 (−0.04–0.03)
Obesity	1.42 (1.15–1.76) **	0.52 (0.30–0.74) ***
Depression	1.38 (1.04–1.83) *	0.02 (−0.28–0.31)
Use of psychotropic medications	1.29 (1.01–1.65) *	−0.44 (−0.70–−0.19) **
Epilepsy/seizure disorder	1.51 (1.16–1.97) **	−0.19 (−0.46–0.08) ^¥^
Urinary incontinence	2.50 (1.73–3.62) ***	−0.63 (−0.96–−0.29) ***
Falls	1.61 (1.26–2.06) ***	−0.04 (−0.29–0.21)
Mobility limitation	4.66 (3.22–6.75) ***	−0.22 (−0.52–0.08) ^¥^
**Social–Environmental Factors**		
Day/educational program or employment (No)	1.27 (0.94–1.74) ^¥^	0.99 (0.67–1.30) ***
Residential type		
Own home (ref)		
Family home	0.76 (0.60–0. 96) *	0.13 (−0.12–0.37)
Foster/group home	1.51 (1.06–2.14) *	−0.61 (−0.96–−0.27) **
Residential location		
Urban	0.80 (0.60–1.06) ^¥^	0.09 (−0.22–0.39)
Social participation	0.86 (0.83–0.89) ***	−0.01 (−0.05–0.02)
Special Olympics involvement	0.80 (0.76–0.85) ***	0.01 (−0.05–0.07)

LLPA = low levels of physical activity; ID = intellectual disability; *OR* = odds ratio; *B* = unstandardized beta coefficient; *CI* = confidence interval. ref = a reference group. Absence of depression, epilepsy/seizure disorder, urinary incontinence, falls (in the past 12 months), and mobility limitation are reference groups. Rural area is a reference group. Informant-rated health status: a higher value indicates better health. A cut-off point of *p* < 0.20 was used to include the variables in the final multiple logistic regression models. ^¥^ *p* < 0.20, * *p* < 0.05, ** *p* < 0.01, *** *p* < 0.001.

**Table 3:** Summary of multiple logistic regressions for LLPA.

Variables	Model I	Model II
	*AOR* (95% *CI*) *N* = 926	*AOR* (95% *CI*) *N* = 1228
**Demographic Factors**		
Age (years)		
18–39 (ref)		
40–59	1.18 (0.82–1.69)	1.30 (0.96–1.76)
≥60	1.77 (0.81–3.90)	2.10 (1.12–3.92) *
Sex (male)	0.70 (0.52–0.95) *	0.78 (0.61–1.01)
ID related conditions		
ID only/others (ref)		
Down syndrome	0.92 (0.64–1.31)	0.93 (0.68–1.27)
Cerebral palsy	1.93 (1.09–3.44) *	1.94 (1.21–3.13) **
Autism spectrum disorder	0.79 (0.48–1.31)	0.85 (0.56–1.27)
Level of ID		-
Borderline (ref)		
Mild or moderate	0.96 (0.65–1.41)	
Severe or profound	1.29 (0.68–2.43)	
**Health and Function Factors**		
Informant-rated health status	0.72 (0.59–0.89) **	0.80 (0.68–0.95) *
Days of activity limitation	1.04 (0.99–1.08)	1.04 (1.01–1.08) *
Number of chronic conditions	0.98 (0.91–1.05)	0.97 (0.91–1.03)
Obesity	1.60 (1.17–2.19) **	1.58 (1.21–2.06) **
Depression	1.67 (1.02–2.71) *	1.27 (0.84–1.93)
Use of psychotropic medications	1.00 (0.67–1.49)	0.98 (0.70–1.39)
Epilepsy/seizure disorder	1.06 (0.71–1.60)	1.08 (0.76–1.52)
Urinary incontinence	1.06 (0.57–1.98)	1.04 (0.62–1.74)
Falls	1.01 (0.69–1.47)	0.95 (0.69–1.30)
Mobility limitation	2.34 (1.23–4.47) *	2.32 (1.41–3.80) **
**Social-Environmental Factors**		
Day/educational program or employment (No)	0.60 (0.36–0.98) *	0.68 (0.45–1.02)
Residential type		
Own home (ref)		
Family home	0.92 (0.63–1.33)	1.11 (0.81–1.52)
Foster/group home	0.84 (0.50–1.41)	1.14 (0.73–1.78)
Residential location		
Urban	0.81 (0.52–1.27)	0.97 (0.68–1.38)
Social participation	0.86 (0.82–0.91) ***	0.88 (0.84–0.92) ***
Special Olympics involvement	0.95 (0.86–1.03)	0.92 (0.85–0.98) *

LLPA = low levels of physical activity; ID = intellectual disability; *AOR* = adjusted odds ratio; *CI* = confidence interval. ref = a reference group. Absence of depression, epilepsy/seizure disorder, urinary incontinence, falls (in the past 12 months), and mobility limitation are reference groups. Rural area is a reference group. Informant-rated health status: a higher value indicates better health. * *p* < 0.05, ** *p* < 0.01, *** *p* < 0.001.

**Table 4:** Summary of multiple regressions for TV-watching time.

Variables	Model I	Model II
	*B* (95% *CI*) *N* = 970	*B* (95% *CI*) *N* = 1292
**Demographic Factors**		
Age (years)		
18–39 (ref)		
40–59	0.01 (−0.29–0.31)	−0.02 (−0.28–0.25)
≥60	0.43 (−0.15–1.01)	0.48 (−0.02–0.96)
Sex (male)	0.34 (0.09–0.59) **	0.31 (0.08–0.53) **
ID related conditions		
ID only/others (ref)		
Down syndrome	−0.03 (−0.34–0.29)	−0.10 (−0.39–0.18)
Cerebral palsy	0.20 (−0.23–0.63)	0.13 (−0.25–0.52)
Autism spectrum disorder	−0.39 (−0.83–0.04)	−0.24 (−0.61–0.13)
Level of ID		
Borderline (ref)		
Mild or moderate	−0.26 (−0.60–0.08)	
Severe or profound	−0.76 (−1.26–−0.26) **	
**Health and Function Factors**		
Informant-rated health status	−0.11 (−0.28–0.05)	−0.08 (−0.23–0.06)
Days of activity limitation	−0.01 (−0.02–0.04)	−0.00 (−0.03–0.02)
Obesity	0.48 (0.21–0.75) ***	0.44 (0.20–0.68) ***
Use of psychotropic medications	−0.21 (−0.52–−0.11)	−0.30 (−0.59–−0.02) *
Epilepsy/seizure disorder	−0.35 (−0.68–0.01) *	−0.30 (−0.60–0.00)
Urinary incontinence	−0.37 (−0.83–0.10)	−0.33 (−0.73–0.08)
Mobility limitation	−0.29 (−0.73–0.15)	−0.23 (−0.60–0.15)
**Social-Environmental Factors**		
Day/educational program or employment (No)	1.39 (0.98–1.79) ***	1.14 (0.78–1.49) ***
Residential type		
Own home (ref)		
Family home	0.35 (0.03–0.66) *	0.25 (−0.03–0.53)
Foster/group home	−0.51 (−0.92–−0.09) *	−0.46 (−0.83–−0.08) *

ID = intellectual disability; *B* = unstandardized beta coefficient; *CI* = confidence interval. ref = a reference group. Absence of depression, epilepsy/seizure disorder, urinary incontinence, falls (in the past 12 months) and mobility limitation are reference groups. Being in a day/educational program or employment is a reference group. Rural area is a reference group. Informant-rated health status: a higher value indicates better health. * *p* < 0.05, ** *p* < 0.01, *** *p* < 0.001.

## References

[1] LaMonte M.J., Barlow C.E., Jurca R., Kampert J.B., Church T.S., Blair S.N. (2005). Cardiorespiratory Fitness Is Inversely Associated With the Incidence of Metabolic Syndrome; A Prospective Study of Men and Women. Circulation.

[2] Laaksonen D.E., Lakka H.M., Salonen J.T., Niskanen L.K., Rauramaa R., Lakka T.A. (2002). Low Levels of Leisure-Time Physical Activity and Cardiorespiratory Fitness Predict Development of the Metabolic Syndrome. Diabetes Care.

[3] World Health Organization (2009). Global Health Risks: Mortality and Burden of Disease Attributable to Selected Major Risks.

[4] Dogra S., Stathokostas L. (2012). Sedentary behavior and physical activity are independent predictors of successful aging in middle-aged and older adults. J. Aging Res..

[5] De Rezende L.F.M., Lopes M.R., Rey-López J.P., Matsudo V.K.R., do Carmo Luiz O. (2014). Sedentary behavior and health outcomes: An overview of systematic reviews. PLoS ONE.

[6] Wilmot E.G., Edwardson C.L., Achana F.A., Davies M.J., Gorely T., Gray L.J., Khunti K., Yates T., Biddle S.J. (2012). Sedentary time in adults and the association with diabetes, cardiovascular disease and death: Systematic review and meta-analysis. Diabetologia.

[7] Patel A.V., Bernstein L., Deka A., Feigelson H.S., Campbell P.T., Gapstur S.M., Colditz G.A., Thun M.J. (2010). Leisure time spent sitting in relation to total mortality in a prospective cohort of US adults. Am. J. Epidemiol..

[8] Tremblay M.S., Aubert S., Barnes J.D., Saunders T.J., Carson V., Latimer-Cheung A.E., Chastin S.F., Altenburg T.M., Chinapaw M.J. (2017). Sedentary Behavior Research Network (SBRN)—Terminology Consensus Project process and outcome. Int. J. Behav. Nutr. Phys. Act..

[9] Hamilton M.T., Hamilton D.G., Zderic T.W. (2007). Role of low energy expenditure and sitting in obesity, metabolic syndrome, type 2 diabetes, and cardiovascular disease. Diabetes.

[10] Healy G.N., Dunstan D.W., Salmon J.O., Shaw J.E., Zimmet P.Z., Owen N. (2008). Television time and continuous metabolic risk in physically active adults. Med. Sci. Sports Exerc..

[11] Owen N., Healy G.N., Matthews C.E., Dunstan D.W. (2010). Too Much Sitting: The Population Health Science of Sedentary Behavior. Exerc. Sport Sci. Rev..

[12] Chastin S.F., De Craemer M., Lien N., Bernaards C., Buck C., Oppert J.M., Nazare J.A., Lakerveld J., O’Donoghue G., Holdsworth M. (2016). The SOS-framework (Systems of Sedentary behaviours): An international transdisciplinary consensus framework for the study of determinants, research priorities and policy on sedentary behaviour across the life course: A DEDIPAC-study. Int. J. Behav. Nutr. Phys. Act..

[13] Draheim C.C., Williams D.P., McCubbin J.A. (2002). Prevalence of physical inactivity and recommended physical activity in community-based adults with mental retardation. Ment. Retard..

[14] Hilgenkamp T.I., Reis D., van Wijck R., Evenhuis H.M. (2012). Physical activity levels in older adults with intellectual disabilities are extremely low. Res. Dev. Disabil..

[15] Robertson J., Emerson E., Gregory N., Hatton C., Turner S., Kessissoglou S., Hallam A. (2000). Lifestyle related risk factors for poor health in residential settings for people with intellectual disabilities. Res. Dev. Disabil..

[16] Oviedo G.R., Travier N., Guerra-Balic M. (2017). Sedentary and Physical Activity Patterns in Adults with Intellectual Disability. Int. J. Environ. Res. Public Health.

[17] 2008 Physical Activity Guidelines for Americans. http://www.health.gov/paguidelines/pdf/paguide.pdf.

[18] Peterson J.J., Janz K.F., Lowe J.B. (2008). Physical activity among adults with intellectual disabilities living in community settings. Prev. Med..

[19] Barnes T.L., Howie E.K., McDermott S., Mann J.R. (2013). Physical activity in a large sample of adults with intellectual disabilities. J. Phys. Act. Health.

[20] Dairo Y.M., Collett J., Dawes H., Oskrochi G.R. (2016). Physical activity levels in adults with intellectual disabilities: A systematic review. Prev. Med. Rep..

[21] Hsieh K., Heller T., Bershadsky J., Taub S. (2015). Impact of Adulthood Stage and Social-Environmental Context on Body Mass Index and Physical Activity of Individuals with Intellectual Disability. Intellect. Dev. Disabil..

[22] Stancliffe R.J., Anderson L.L. (2017). Factors associated with meeting physical activity guidelines by adults with intellectual and developmental disabilities. Res. Dev. Disabil..

[23] Finlayson J., Jackson A., Cooper S.A., Morrison J., Melville C., Smiley E., Allan L., Mantry D. (2009). Understanding predictors of low physical activity in adults with intellectual disabilities. J. Appl. Res. Intellect. Disabil..

[24] Melville C.A., Boyle S., Miller S., Macmillan S., Penpraze V., Pert C., Spanos D., Matthews L., Robinson N., Murray H. (2011). An open study of the effectiveness of a multi-component weight-loss intervention for adults with intellectual disabilities and obesity. Br. J. Nutr..

[25] Mikulovic J., Vanhelst J., Salleron J., Marcellini A., Compte R., Fardy P.S., Bui-Xuan G. (2014). Overweight in intellectually-disabled population: Physical, behavioral and psychological characteristics. Res. Dev. Disabil..

[26] Hsieh K., Rimmer J.H., Heller T. (2014). Obesity and associated factors in adults with intellectual disabilities. J. Intellect. Disabil. Res..

[27] Frey G.C. (2004). Comparison of physical activity levels between adults with and without mental retardation. J. Phys. Act. Health.

[28] Temple V., Walkley J.W. (2003). Physical activity of adults with intellectual disability. J. Intellect. Dev. Disabil..

[29] Nordstrøm M., Hansen B.H., Paus B., Kolset S.O. (2013). Accelerometer-determined physical activity and walking capacity in persons with Down syndrome, Williams syndrome and Prader-Willi syndrome. Res. Dev. Disabil..

[30] Izquierdo-Gómez R., Martínez-Gómez D., Acha A., Veiga O.L., Villagra A., Diaz-Cueto M., UP&DOWN Study Group (2014). Objective assessment of sedentary time and physical activity throughout the week in adolescents with Down syndrome. The UP&DOWN study. Res. Dev. Disabil..

[31] Chastin S.F., Buck C., Freiberger E., Murphy M., Brug J., Cardon G., O’Donoghue G., Pigeot I., Oppert J.M. (2015). Systematic literature review of determinants of sedentary behaviour in older adults: A DEDIPAC study. Int. J. Behav. Nutr. Phys. Act..

[32] Basterra-Gortari F.J., Bes-Rastrollo M., Gea A., Núñez-Córdoba J.M., Toledo E., Martínez-González M.Á. (2014). Television Viewing, Computer Use, Time Driving and All-Cause Mortality: The SUN Cohort. J. Am. Heart Assoc..

[33] Anderson S., Currie C.L., Copeland J.L. (2016). Sedentary behavior among adults: The role of community belonging. Prev. Med. Rep..

[34] Keadle S.K., Conroy D.E., Buman M.P., Dunstan D.W., Matthews C.E. Targeting Reductions in Sitting Time to Increase Physical Activity and Improve Health. Med. Sci. Sports Exerc..

[35] Young D.R., Hivert M.F., Alhassan S., Camhi S.M., Ferguson J.F., Katzmarzyk P.T., Lewis C.E., Owen N., Perry C.K., Siddique J. (2016). Sedentary Behavior and Cardiovascular Morbidity and Mortality: A Science Advisory From the American Heart Association. Circulation.

[36] Hsieh K., Rimmer J., Heller T. (2012). Prevalence of falls and risk factors in adults with intellectual disability. Am. J. Intellect. Dev. Disabil..

[37] Tinetti M.E., Baker D.I., Dutcher J., Vincent J.E., Rozett R.T. (1997). Reducing the Risk of Falls among Adults in the Community.

[38] Hosmer D.W., Lemeshow S. (2000). Applied Logistic Regression.

[39] Bendel R.B., Afifi A.A. (1977). Comparison of stopping rules in forward regression. J. Am. Stat. Assoc..

[40] Mickey J., Greenland S. (1989). A study of the impact of confounder selection criteria on effect estimation. Am. J. Epidemiol..

[41] Fox J., Monette G. (1992). Generalized Collinearity Diagnostics. J. Am. Stat. Assoc..

[42] R Core Team (2017). R: A Language and Environment for Statistical Computing.

[43] IBM Corp (2016). IBM SPSS Statistics for Windows.

[44] Dixon-Ibarra A., Lee M., Dugala A. (2013). Physical activity and sedentary behavior in older adults with intellectual disabilities: A comparative study. Adapt. Phys. Act. Q..

[45] Rimmer J.H. (2000). Achieving a Beneficial Fitness: A Program and a Philosophy in Mental Retardation.

[46] Marks B., Sisirak J., Chang Y.C. (2013). Efficacy of the Health Matters Program Train-the-Trainer Model. J. Appl. Res. Intellect. Disabil..

[47] Heller T., Hsieh K., Rimmer J.H. (2004). Attitudinal and psychosocial outcomes of a fitness and health education program on adults with down syndrome. Am. J. Ment. Retard..

[48] Bergström H., Hagströmer M., Hagberg J., Elinder L.S. (2013). A multi-component universal intervention to improve diet and physical activity among adults with intellectual disabilities in community residences: A cluster randomised controlled trial. Res. Dev. Disabil..

